# Barriers to Adherence to Healthy Diet and Recommended Physical Activity Perceived by the Polish Population

**DOI:** 10.3390/jcm13010022

**Published:** 2023-12-19

**Authors:** Katarzyna Domosławska-Żylińska, Magdalena Łopatek, Magdalena Krysińska-Pisarek, Larysa Sugay

**Affiliations:** 1 Department of Education and Communication, National Institute of Public Health NIH—National Research Institute, 24 Chocimska St., 00-791 Warsaw, Poland; mlopatek@pzh.gov.pl (M.Ł.); mkrysinska@pzh.gov.pl (M.K.-P.); 2 Centre for Migration Studies, Adam Mickiewicz University in Poznań (CeBaM AMU), 7 Uniwersytetu Poznańskiego St., 61-614 Poznań, Poland; larsug@amu.edu.pl

**Keywords:** barriers, healthy lifestyle, physical activity, healthy diet, health promotion

## Abstract

Background: According to the World Health Organization, an unhealthy diet and lack of physical activity constitute the primary global health risks. The purpose of this study was to as-certain the barriers to a healthy diet (HD) and physical activity (PA) as perceived by the Polish population in order to implement public health interventions. Methods: A quantitative survey was conducted using the computer-assisted telephone interview technique on a randomly selected representative sample of 2000 Polish citizens aged 18–88 years. The research tool was a questionnaire consisting of two parts: sociodemographic characteristics and examining barriers to an HD (Cronbach’s alpha = 0.899) and regular PA (Cronbach’s alpha = 0.923). Results: Women constituted more than half of the sample (53.4%), and most of the respondents lived in urban areas (60.5%), considered their financial situation as average (56.9%), and their health as satisfactory (42.3%). Barriers to an HD include the cost of healthy food (43%), lack of motivation (26.7%), and lack of time (25.4%). Barriers to taking up PA include competing priorities (29%), a lack of motivation to exercise (27.3%), feeling of constant fatigue, and lack of energy (24.4%). Limiting factors in the adoption of both an HD and PA are gender (women > men; HD *p* < 0.01; PA *p* < 0.001), financial situation (unsatisfactory; HD and PA *p* < 0.001), health condition (unsatisfactory; HD and PA *p* < 0.001), type of work (blue-collar workers; HD *p* < 0.001; PA *p* < 0.05), and employment status (people running household; HD and PA *p* < 0.001). Conclusions: The results of this study provide important information about barriers to adopting healthy lifestyle principles. The practical implications of our work can be used by policymakers responsible for intervention strategies and programmes to increase the number of people adhering to recommendations for an HD and PA by removing barriers.

## 1. Introduction

According to the World Health Organization (WHO), an unhealthy diet and lack of physical activity constitute major global health risks [1]. A healthy diet (HD) and physical activity (PA) are associated with numerous health benefits, preventing a number of non-communicable diseases which account for almost three-quarters of deaths (41 million) worldwide: hypertension, obesity, heart disease, stroke, diabetes, and certain types of cancer. Additionally, it can significantly improve mental health, quality of life, and overall well-being [2,3,4].

Recommendations for a healthy diet are based on the Healthy Eating Plate (half of the portion on the plate should be vegetables and fruits, a quarter grain products, and a quarter products that are a source of protein) and include, among others, reducing the intake of salt, red meat and processed meat products, simple refined sugars and sweetened drinks, and highly processed products, and increasing the intake of various vegetables and fruits, whole grain cereal products, legumes, fish, low-fat dairy products (milk, yoghurt, kefir, buttermilk, cottage cheese), nuts, and seeds [5].

According to current Polish recommendations, adults (18–64 years old) should undertake at least 150–300 min of moderate-intensity aerobic physical activity per week; or at least 75–150 min of high-intensity aerobic physical activity; or an equivalent combination of moderate- and high-intensity activities. Above that there is a recommendation of performing moderate- or higher-intensity muscle-strengthening exercises, covering all major muscle groups, at least two days per week [6].

The increased availability and prevalence of processed food consumption, escalating urbanization, lifestyle changes, the adoption of sedentary forms of transportation, and the utilization of technology for leisure activities have all altered dietary patterns and reduced physical activity [7,8,9]. A notable surge in the intake of high-energy foods, fats, free sugars, and salt/sodium, coupled with insufficient consumption of fruit, vegetables, and whole grain products has been reported. Concurrently, physical activity statistics show that one in four adults do not undertake the recommended amount of physical activity [7].

Statistics pertaining to the Polish population show that the majority of people (61.2%) do not undertake physical activity [10]. According to the recent Organization for Economic Cooperation and Development (OECD)/WHO report, Poland (about 20% of people undertaking exercise and sport at least once a week) ranks notably below the European average (40% of people undertaking exercises and sport at least once a week) concerning the level of physical activity [11]. In terms of adherence to healthy eating habits, it was reported that only 24% of Polish society followed dietary recommendations in 2022 [12]. In recent years, there has been a discernible increase in the consumption of fats, especially butter, sugar, confectionery, and salt [12].

The data presented above indicate a considerable gap between the expected frequency of behaviours related to a healthy diet or physical activity and actual behaviours. A gap in knowledge becomes visible, prompting questions about the origins of these discrepancies and why people do not follow the recommendations for a healthy lifestyle. Due to the complexity of the topic, the scientific literature discusses potential factors—individual, environmental, and economic—that may hinder healthy behaviours, possibly contributing to the observed discrepancies [13].

In models and theories of behavioural change or health promotion, the above-mentioned potential barriers limiting change or following recommendations are one of the key variables. Pender’s Health Promotion Model defines barriers to healthy lifestyles as factors that directly interfere with the implementation of health-promoting behaviours or contribute, through reduced commitment to an action plan, towards behavioural change [14].

The scientific literature reveals that the barriers hindering the adopting of healthy lifestyles exhibit variations across different countries and societies. This can be partially attributed to cultural differences, socio-economic factors, and political conditions [15]. The limiting factors, also considered as barriers to an HD and PA, can be classified both as internal and external. For example, external barriers are related to the environment, e.g., access to infrastructure. The identified internal barriers encompass personal and interpersonal factors such as willpower, self-regulation, and the influence of family and peers, as well as lack of resources or time [16,17]. Research indicates that approximately 80% of Polish society considers issues related to healthy eating as important or very important, while 40% find maintaining a healthy diet challenging [18]. The above mentioned information highlights a gap, and underscores the need to supplement knowledge regarding factors that make it difficult to undertake health-beneficial behaviours related to an HD at the individual level. Factors influencing the adoption of an HD include a range of aspects, with dietary choices being contingent on many factors, such as taste preferences, ease of obtaining healthy products, knowledge, traditions, and cultural habits [19]. The most frequently mentioned barriers related to healthy eating in the literature included a lack of money and time for preparing and consuming nutritious meals, belief that the recommended amount of vegetables is too large, lack of enjoyment from eating healthy food, and insufficient nutritional knowledge [17].

Research results have indicated that individual barriers are among the primary predictors of behaviours related to physical activity and sports participation [20]. Barriers associated with PA may deter individuals from engaging in physical activity and promote a sedentary lifestyle. Cultural and social patterns, as well as the value attributed by the environment to an active lifestyle play a significant role in influencing activity uptake [15]. The most frequently mentioned barriers regarding PA included lack of time, easy access to sedentary behaviours, negative experiences related to the practice of physical activity, and issues with mobility and health [17]. In the case of barriers related to physical activity, there is scientific evidence confirming the importance of limiting factors related to the external environment, but there remains a knowledge gap concerning internal, subjective barriers. There is currently no research in Poland that examines the barriers to an HD and PA perceived by the adult population.

The aim of the study is to identify barriers perceived by the Polish society, to verify whether and which groups of respondents declare greater impediments to implementing healthy lifestyle recommendations, and also to compare which barriers are most burdensome for each group of respondents. This study is undertaken in order to guide and enhance the design and implementation of evidence-based interventions aimed at fostering the improvement of behavioural changes conducive to improving healthy lifestyles [16].

## 2. Materials and Methods

### 2.1. Study Participants

A representative sample of 2000 respondents who were Polish citizens aged 18 and above participated in the study. The representative sample was calculated using a sample selection calculator, with the following assumptions: population size of 37,698,000 people according to the Central Statistical Office (June 2023), a confidence level of 98%, and a maximum error of 3%.

### 2.2. Procedures

The quantitative survey was conducted in November 2022 using the computer-assisted telephone interview technique (CATI). Random selection was used to conduct the survey. A total of 2119 people were randomly selected for the study, which ultimately resulted in 2000 correctly completed questionnaires. The survey tool was an opinion poll panel, belonging to Biostat, Warsaw, Poland. A sampling frame was used to initiate contact with respondents, which included a database of contact numbers, including both landline and mobile phone numbers operating in Poland. The sociodemographic data were verified using the survey’s inclusion (metric) questions. The respondents took part in the survey based on their informed verbal consent. Before the survey, they were informed about the purpose of the study, the data anonymization, the scientific nature of the application of the results, and the possibility to withdraw from the study at any time.

### 2.3. Research Tool

The research questionnaire consisted of two sections: the first encompassed sociodemographic information (12 questions), while the second section focused on potential barriers associated with the implementation of healthy lifestyle principles. The barriers were selected on the basis of a scoping review. This section further delved into two aspects: a healthy diet (14 statements, e.g., costs of healthy food; Cronbach’s alpha = 0.899) and regular physical activity (16 statements, e.g., lack of time and competing priorities (work, family, hobbies); Cronbach’s alpha = 0.923). A healthy diet was defined as consumption of regular, diverse meals, rich in complex carbohydrates, legumes, vegetables, and fruit, while minimizing or avoiding excessive amounts of animal fat, salt, sweets, highly processed foods, and alcohol. Regular physical activity was operationally defined as activity performed at least three times a week, amounting to 150–300 min per week. Respondents answered the question: “to what extent do the following factors limit your ability to follow a healthy diet/physical activity on a daily routine”. Answers were given on the following scale: 1—definitely limiting, 2—limiting to a small extent, 3—not really limiting, 4—definitely not limiting.

The survey questionnaire was pilot-tested with 5 substantive employees of the institute (NIPH NIH—NRI) and 5 non-content employees as representatives of the general population. Based on the pilot study, improvements were made to the questionnaire to increase comprehension and readability.

### 2.4. Statistical Analysis

The summary (average) of the Healthy Eating Barriers Index and the Physical Activity Barriers Index global were calculated for each group of respondents in each block of questions. A higher value of each index corresponds to a greater restriction for respondents in following recommendations for a healthy diet or physical activity. Other statistical tests included a chi-square test, which was used to determine the independence of the two categorical variables. For the relationship between a quantitative variable and a qualitative variable, due to the lack of a normal distribution among the quantitative variables studied, non-parametric Mann–Whitney U tests (for two groups) or Kruskal–Wallis tests (three or more groups) were used. Tables with barriers limiting a healthy diet and physical activity depending on factors such as age, gender, level of education, status on the labour market, the place of residence, and type of work performed, labelled S1–S13, can be found in the Supplementary Material. The significance level was established at 0.05 and *p*-values were presented as consecutive significance levels: *p* < 0.05, *p* < 0.01, and *p* < 0.001. Data analysis was performed using the R software (version 4.0.0) or Microsoft Excel (version 2311).

## 3. Results

The survey included 2000 respondents, more than half of them were women (53.4%), with the youngest participant being 18 years old and the oldest 88 years old. Most of the respondents (60.5%) lived in urban areas, had secondary or post-secondary education (42.5%), and were employed (62.6%). White-collar work was performed by approximately 44.8% of the respondents. More than half of the respondents (56.9%) stated that their financial situation was average. A total of 42.3% of the respondents considered their health as satisfactory (Table 1).

### 3.1. Barriers to Healthy Diet

The analysis of the responses regarding barriers to healthy eating (Table 2) indicates that the major impediment to adhering to and following a healthy diet is the cost of healthy food. Among the barriers described as definitely limiting, respondents also mentioned deficiency or lack of motivation to enact changes, lack of time, and competing priorities, such as work, family, or hobbies, and the necessity to adjust existing habits to a healthier diet. These factors listed above were deemed as definitely limiting by more than 25% of the respondents.

#### Analysis of Sociodemographic Factors Influencing Barriers to Healthy Eating on a Daily Basis

The Healthy Eating Barrier Index for gender was more pronounced in women than in men (Table 3). Women were more likely than men to indicate the limiting nature of the following factors in hindering a healthy diet: the cost of healthy food, no or difficult access to good-quality products from organic farming and breeding, lack of knowledge on current healthy eating recommendations, lack of support from family and friends, and lack of support, solutions, or proposals from the healthcare system (primary care physicians, dietitians, physiotherapists). Detailed information is presented in Table S1 of the Supplementary Material.

The Healthy Eating Barrier Index based on age reached its highest value among people in the age range of 30–39 years old. These people were more inclined than those in other age groups to indicate the limiting nature of the assessed barriers, such as lack of time and competing priorities like work, family, and hobbies (Table S2). When analysing the factor of place of residence, it was observed that the Healthy Eating Barrier Index exhibited its highest value among rural residents.

The Healthy Eating Barrier Index for education peaked among respondents with primary education. That group of individuals demonstrated a greater likelihood than respondents at other educational levels to indicate the limiting nature of the identified barriers such as deficiency in knowledge and skills pertaining to steps that need to be taken in order to apply healthy lifestyle principles, and lack of awareness regarding current healthy eating recommendations (Table S3).

The Healthy Eating Barriers Index based on employment status reached the highest value among people running a household. These individuals were more likely than other respondents with other employment statuses to highlight the limiting nature of the healthy eating barriers in question, including the absence of support, solutions, or proposals from the healthcare system (primary care physicians, dietitians, physiotherapists), and lack of belief in the efficacy of a healthy diet for preventing lifestyle-related diseases) (Table S4).

The Healthy Eating Barriers Index associated with financial situation recorded its peak value among people declaring themselves to be in an unsatisfactory financial situation. Nearly all of the discussed barriers, with the exception of “lack of time” and “competing priorities”, were more frequently perceived as limiting by respondents indicating their unsatisfactory financial situation than those reporting a satisfactory or average financial situation (Table S5).

The Healthy Eating Barriers Index for individual’s health condition reached the highest value among those survey participants who declared an unsatisfactory health condition. These people were more likely than other respondents to indicate the limiting nature of the barriers in question, with the exception of “lack or limited access to good-quality, organically grown and bred products” (Table S6).

The Healthy Eating Barriers Index for the type of work performed reached the highest value among blue-collar workers. These people were more likely than those in mixed or white-collar jobs to point to the limiting nature of the barriers in question such as lack of motivation to implement changes, lack of time and competing priorities (work, family, hobbies), lack of knowledge and skills with regard to steps that need to be taken to apply healthy lifestyle principles, lack of belief in the efficacy of a healthy diet for preventing lifestyle-related diseases, lack of knowledge on current healthy eating recommendations, and the taste of healthy food (Table S7).

### 3.2. Barriers Limiting the Ability to Undertake Regular Physical Activity

Lack of time and competing priorities were most frequently indicated by the respondents as factors hindering physical activity. The next most frequently mentioned barrier was a lack of motivation to exercise, followed by a lack of willingness to undertake physical activity and a feeling of constant fatigue or lack of energy (Table 4).

#### Analysis of Sociodemographic Factors Influencing Barriers to Engaging in Regular Physical Exercise

The Physical Activity Barriers Index for gender reached a higher value for women compared to men (Table 5). Women were more inclined than men to indicate the limiting nature of the following factors hindering their regular physical activity: barriers in the built-up environment, adverse weather conditions, insufficient support from the healthcare system and family, lack of willingness and motivation to exercise, persistent fatigue, lack of appropriate skills, and lack of belief in the effectiveness of physical activity in the prevention of lifestyle-related diseases (Table S8).

The Physical Activity Barrier Index for the place of residence attained its highest value among rural residents. Rural residents were more prone than their urban counterparts to indicate the limiting nature of specific barriers, notably barriers related to the built-up environment and geographical isolation such as the absence of suitable exercise locations, lack of access to fitness clubs and pools, as well as a lack of belief in the effectiveness of physical activity in preventing lifestyle-related diseases (Table S9).

The Physical Activity Barrier Index based on employment status reached the highest value within the group of individuals responsible for running a household. These individuals were more likely than respondents with a different employment status to highlight the constraining nature of barriers, including competition with activities (such as watching TV), lack of support from family and friends (having no one to exercise with), and barriers in the built-up environment and geographical isolation, such as the absence of suitable exercise locations, and lack of access to fitness clubs and pools (Table S10).

The Physical Activity Barrier Index associated with financial situation peaked among individuals who declared themselves to be in an unsatisfactory financial situation. These individuals were more inclined to indicate almost all barriers as hindrances to engaging in physical activity, with the exception of the issue related to a lack of time and competing priorities (work, family, hobbies) (Table S11).

The Physical Activity Barrier Index for health condition reached its highest value among respondents who declared an unsatisfactory health condition. They were more likely than those declaring average or satisfactory health to highlight the limiting nature of all barriers in question (Table S12).

The Physical Activity Barrier Index based on employment status reached the highest value in the group of people engaged in blue-collar work. These people were more likely than those in mixed or white-collar jobs to indicate the limiting nature of the barriers, particularly lack of belief in the effectiveness of physical activity in preventing lifestyle-related diseases (Table S13).

## 4. Discussion

As delineated, there is a knowledge gap pertaining to the factors that contribute to disparities between HD and PA recommendations and the tangible behavioural choices made by the population in Poland. Hence, this study was initiated to address the existing knowledge gap concerning the subjective observations of the Polish society regarding factors that impede adherence to HD and PA recommendations.

The cost of healthy food, recognised as a barrier to healthy eating, and a lack of time and motivation, which apply to both healthy eating and physical activity, were identified as the most limiting factors that prevent compliance with guidelines for a healthy lifestyle. The financial aspect (cost of a diet) plays a pivotal role in influencing food choices, determining diet quality and affecting food security. The elevated cost of healthy food leads to decreased nutrient intake, resulting in suboptimal diet and micronutrient deficiencies [21]. In 2021, more than three billion people, constituting 42% of the world’s population, were unable to afford a healthy diet. Notably, food of animal origin is the most expensive food group, while vegetables rank as the second most expensive [22]. Variations in food expenditure can be found in various studies. Some researchers indicate that maintaining a healthy diet is associated with higher total expenditure, others that adhering to a healthy diet might be more economical than adopting an unhealthy one [23]. The recently observed 19% increase in food prices in Poland compared to the previous year may be conducive to a lower diet quality and lead to inadequate consumption of nutrient-rich foods [24]. Improving the affordability of healthy foods has the potential to enhance the quality of the population’s diet [25]. Time pressure as a barrier to healthy eating is observed among those working more than 40 h per week and is associated with a lower consumption of fruit and vegetables and higher fast food intake [26,27]. In contrast, lack of time (perceived or actual) as a barrier to physical activity is associated with the belief that other responsibilities are more important and require commitment, thus discouraging engagement in physical activity. Lack of motivation, defined as a barrier applicable to both a healthy diet and physical activity, requires an individualized approach to improve adherence [28,29]. Additionally, extrinsic motivation, involving regular contact with an expert (in terms of diet and physical activity) helps to maintain health-promoting behaviours [13].

Limiting factors in the adoption of both a healthy diet and physical activity are gender (female), financial situation (unsatisfactory), type of employment (running household), health condition (unsatisfactory), and type of work (blue-collar). Moreover, age (specifically, the range of 30–39 years) and education (primary level) were identified as barriers for a healthy diet, while place of residence (specifically, rural areas) was flagged as a limiting factor for physical activity. Individuals from distinct demographic groups exhibited variations in their perceived barriers. Recognising the identified barriers specific for given groups may be useful for policymakers and practitioners, and help to optimise healthy lifestyle interventions by strategically addressing and overcoming identified constraints [13].

For Polish women, the biggest limiting factors to adopting a healthy lifestyle include the costs of healthy food, lack of support from the healthcare system and family (pertaining to both physical activity and healthy diet), lack of willingness and motivation (physical activity), feeling of constant fatigue (physical activity), and difficulty in accessing sports facilities (physical activity). As research shows, a healthy diet and physical activity, constituting elements of a healthy lifestyle, are not always a priority for women, especially those of lower economic status [30].

From the perspective of women, according to their declarations, there is a notable concern pertaining to physical appearance, which may potentially culminate in persistent discontent with their body shape or weight. This, in turn, can lead to feelings of embarrassment, diminished self-esteem, a perception of complete lack or limited influence over circumstances, and consequently, constraints on the adoption of PA [31]. Experiencing adverse societal behaviour, particularly stigmatization directed at overweight women, constitutes a significant factor limiting the inclination to engage in PA [32]. It is undoubtedly worth mentioning the image of a slender female body created and propagated by the media, often unattainable and synonymous with ideals of beauty and health. This depiction has faced escalating criticism due to the negative consequences related to body image, especially among young women [33].

An important factor constraining the level of physical activity among women is the fulfilment of diverse social roles (mother, wife, employee), coupled with the associated pressures [34]. The role of a mother is recognised by society as “natural” and at the same time associated primarily with fulfilling household responsibilities and childcare. Men, in turn, have socially assigned responsibilities related to securing their financial stability. In the case of men, if these expectations are met, they are encouraged to pursue self-fulfilment in their free time, outside the family sphere. However, women who are mothers fulfilling their role more frequently face social judgement when pursuing self-fulfilment, e.g., through PA. This can result in a sense of guilt due to the fact that the role of a mother is perceived as insufficient for them [35,36].

Women emphasize that adhering to healthy lifestyle principles demands routine and self-discipline, and they find family support to be motivating in fostering behaviour change [30]. Psychological factors, including a sense of failure, non-hunger-related eating habits, seeking solace in food, and consuming food in response to stress and emotions, constitute significant barriers to adhering to an HD, particularly for women [37]. The association of an HD with home cooking adds another layer, demanding knowledge, increased effort in planning purchases, and time needed for meal preparation. Involvement in the process of preparing a healthy meal is also perceived by respondents as a barrier due to the burden of responsibilities accumulating from various social roles fulfilled by women [38]. The lack of convenient and affordable access to stores offering healthy food disproportionately affects women, who are typically responsible for the shopping and food preparation process. Consequently, women are more aware of food prices and the disparities between healthy and unhealthy food options available [39,40]. Aspects that are worth highlighting to improve a healthy diet among women include self-efficacy, knowledge, and outcome expectations, encompassing health and appearance benefits [41].

The survey found that an unsatisfactory financial situation was a significant factor influencing individuals’ perceptions of barriers related to physical activity (15 out of 16) and a healthy diet (13 out of 14). Research consistently indicates that people of low socio-economic status are more likely to have poorer health outcomes, exhibiting lower adherence to healthy eating patterns and demonstrating reduced engagement in physical activity. The results of this study, in conjunction with evidence from other studies, indicate the significance of targeting individuals with low socio-economic status as an important target group for implementing lifestyle interventions [42].

In the present study, an unsatisfactory health state constitutes a significant impediment to both physical activity and healthy eating. Some studies show that patients with a negative perception of their health encounter greater challenges in adhering to the recommended guidelines for maintaining a healthy lifestyle [40]. Moreover, among patients with chronic diseases, the reluctance to implement healthy lifestyle principles varies depending on their affliction [39]. In contrast, certain studies demonstrate that an unsatisfactory health state can serve as a motivator or facilitator, as individuals may not prioritize their health when they are in good physical condition [32].

In addition, people with lower education and those in younger age groups experience more restrictions in following a healthy diet compared to people with a higher level of education and those in older age brackets. A lower level of education is associated with a lower level of nutritional knowledge (lack of knowledge on current dietary recommendations, what steps should be taken to apply healthy lifestyle principles, and knowledge of benefits of a healthy diet). A lack of nutritional knowledge has also been identified by other researchers as a key factor limiting healthy eating [43]. The youngest participants in the study cited “easy access to high-calorie and fast-food products” as a limiting factor, while respondents aged 30–39 cited “lack of time and competing priorities”. A young age is often linked to numerous responsibilities, both work- and family-related, while a healthy diet is widely considered to be far more time-consuming than eating ready-made, often highly processed products. Therefore, it is perceived as less convenient [44]. Furthermore, young people tend to attach less importance to healthy eating compared to those at later stages of life [45].

Individuals living in rural areas were more likely than those living in large and small cities to indicate the following factors as limiting: “lack of belief in the effectiveness of physical activity in the prevention of lifestyle-related diseases” and “barriers in the built-up environment and geographical isolation (the absence of suitable exercise locations, lack of access to fitness clubs, pool)”. This highlights the need for implementing educational activities that aim to enhance awareness of the health advantages associated with engaging in physical activity and to suggest activities that are feasible in areas where access to sports facilities is limited. Research findings on the level and availability of physical activity are mixed. Some studies indicate elevated levels of physical activity among people living in medium-sized towns and rural areas when compared to those in both small and large cities [46]. Others indicate existing barriers to physical activity in rural areas, i.e., lack of available instructors, infrastructure, or issues related to social exclusion [47]. Discrepancies in the perception of PA levels may result from variations in the definition of physical activity adopted by respondents. It is plausible that rural residents demonstrate a higher level of spontaneous physical activity related to the use of active forms of transport and essential farm activities [47,48]. The surplus of spontaneous physical activity such as fieldwork, gardening, or taking care of animals may potentially result in a reduced ability to participate in planned or organized forms of PA [49].

The present study featured notable strengths. Firstly, it employed a sample that was representative and sizable, which enhanced the statistical power of the analyses and facilitated generalization of the findings to the broader population. Additionally, the questionnaire used in the study demonstrated a high Cronbach’s alpha coefficient, indicating a high level of reliability for the research tool. However, this study has potential limitations. The data analysed were declarative, relying on self-reported information. Detailed data regarding the reasons for withdrawal from the study were not collected. The study was concentrated on factors limiting the adherence to a healthy diet and physical activity without taking into account contributing factors. This is an area worth exploring in future research. Moreover, it would be beneficial for subsequent studies to investigate the integration of educational resources alongside motivational strategies, as well as the implementation of brief interventions aimed at enhancing self-regulation and promoting healthy behaviours.

## 5. Conclusions

In summation, the results of this study provide important information about barriers to adopting healthy lifestyle principles. Groups that should be given special attention in order to reduce factors limiting compliance with HD and PA recommendations are women, people who assess their financial and health situations as unsatisfactory, manual workers, and people who run the household. The practical implications of our work can be used by policymakers responsible for intervention strategies and programmes to increase the number of people adhering to HD and PA recommendations by removing barriers.

## Figures and Tables

**Table 1:** The characteristics of the respondents (n = 2000; n (%)).

Sociodemographic Characteristics		n (%)
Gender	Women	1047 (52.4)
	Men	953 (47.7)
Age	18–29	322 (16.1)
	30–39	384 (19.2)
	40–59	662 (33.1)
	>60	632 (31.6)
Accommodation	Town ≤ 200,000	592 (29.6)
	Town 200,000–500,000	308 (15.4)
	Town ≥ 500,000	310 (15.5)
	Countryside	790 (39.5)
Education	Primary or middle school	61 (3.1)
	Basic vocational	278 (13.9)
	Secondary or post-secondary education	849 (42.4)
	Higher education (bachelor’s degree and above)	812 (40.6)
Employment	Employed (full-time or self-employed)	1252 (62.6)
	Student	68 (3.4)
	Unemployed	83 (4.2)
	Retired	445 (22.3)
	Disability pensioner	78 (3.9)
	Runs a household	74 (3.7)
Type of work performed	White-collar	896 (44.8)
	Blue-collar	552 (27.6)
	Mixed	552 (27.6)
Self-assessment of financial situation	Very bad	47 (2.4)
	Bad	240 (12.0)
	Average	1138 (56.9)
	Good	515 (25.8)
	Very good	60 (3.0)
Self-assessment of overall health	Very bad	34 (1.7)
	Bad	157 (7.9)
	Average	825 (41.3)
	Good	846 (42.3)
	Very good	138 (6.9)

**Table 2:** Barriers limiting the ability to practise healthy eating on a daily basis (n = 2000; n (%)).

Barriers to HD	Definitely Limiting	Limiting to a Small Extent	Not Really Limiting	Definitely Not Limiting
Costs of healthy food	866 (43.3)	701 (35.1)	353 (17.7)	80 (4.0)
Lack of motivation to enact changes	534 (26.7)	748 (37.4)	511 (25.6)	207 (10.4)
Lack of time and competing priorities (work, family, hobbies)	507 (25.4)	776 (38.8)	479 (24.0)	238 (11.9)
Adjusting habits to a healthier diet	502 (25.1)	880 (44.0)	485 (24.3)	502 (6.7)
Availability of high-calorie and fast-food products	458 (22.9)	689 (34.5)	596 (29.8)	257 (12.9)
Difficulties in avoiding unhealthy foods in local community settings or at gatherings (business, family)	418 (20.9)	799 (40.0)	591 (29.6)	192 (9.6)
Lack of support/solutions/proposals from the healthcare system (primary care physician, dietitian, physiotherapist)	392 (19.6)	710 (35.5)	634 (31.7)	264 (13.2)
Lack or limited access to good-quality and organically grown and bred products	364 (18.2)	771 (38.6)	646 (32.3)	219 (11.0)
Unhealthy or erroneous family dietary patterns	359 (18.0)	702 (35.1)	606 (30.3)	333 (16.7)
Lack of support from family and friends	298 (14.9)	657 (32.9)	698 (34.9)	347 (17.4)
Lack of knowledge and skills regarding the necessary steps to implement healthy lifestyle principles	298 (14.9)	713 (35.7)	669 (33.5)	320 (16.0)
Lack of belief in the efficacy of healthy diet for preventing lifestyle-related diseases	255 (12.8)	671 (33.6)	699 (35.0)	375 (18.8)
Lack of knowledge on current healthy eating recommendations	248 (12.4)	718 (35.9)	704 (35.2)	330 (16.5)
Taste of healthy food	244 (12.2)	705 (35.3)	683 (34.2)	368 (18.4)

**Table 3:** Index of barriers to healthy eating according to sociodemographic factors (n = 2000).

Sociodemographic Factors		M(SD) Index Value	Skewness	Dominant	Kruskal–Wallis	*p*
Gender	Women	2.68 (SD = 0.63)	−0.2	3	533.445 *	*p* < 0.01
	Men	2.60 (SD = 0.59)	−0.3	3		
Age	18–29	2.66 (SD = 0.57)	−0.1	3	10.575	*p* < 0.05
	30–39	2.70 (SD = 0.63)	−0.3	3		
	40–59	2.67 (SD = 0.61)	−0.2	3		
	>60	2.58 (SD = 0.61)	−0.3	3		
Education	Primary or middle school	2.74 (SD = 0.62)	0.0	2.5	8.848	*p* < 0.05
	Basic vocational	2.70 (SD = 0.62)	−0.1	3		
	Secondary or post-secondary education	2.67 (SD = 0.62)	−0.3	3		
	Higher (bachelor’s degree and above)	2.60 (SD = 0.59)	−0.3	3		
Employment status	Working (employed full-time or self-employed)	2.68 (SD = 0.60)	−0.2	3	21.128	*p* < 0.001
	Student	2.56 (SD = 0.51)	−0.1	2.5		
	Unemployed	2.70 (SD = 0.73)	−0.5	3		
	Retired	2.55 (SD = 0.63)	−0.2	3		
	Disability pensioner	2.56 (SD = 0.62)	−0.4	2.1		
	Runs a household	2.79 (SD = 0.61)	−0.2	3.1		
Financial situation	Unsatisfactory	2.81 (SD = 0.64)	−0.4	3	67.400	*p* < 0.001
	Average	2.68 (SD = 0.58)	−0.3	3		
	Satisfactory	2.48 (SD = 0.62)	0.0	2		
Health self-assessment	Unsatisfactory	2.79 (SD = 0.64)	−0.3	3.1	88.251	*p* < 0.001
	Average	2.77 (SD = 0.57)	−0.3	3		
	Satisfactory	2.52 (SD = 0.61)	−0.1	3		
Type of work performed	Blue-collar	2.75 (SD = 0.63)	−0.3	3	23.423	*p* < 0.001
	White-collar	2.59 (SD = 0.54)	−0.2	3		
	Mixed	2.62 (SD = 0.61)	−0.3	3		

* Mann–Whitney U test.

**Table 4:** Barriers limiting the ability to undertake regular physical activity (n = 2000; n (%)).

Barriers to PA	Definitely Limiting	Limiting to a Small Extent	Not Really Limiting	Definitely Not Limiting
Lack of time and competing priorities (work, family, hobbies)	579 (29.0)	710 (35.5)	454 (22.7)	257 (12.9)
Lack of motivation to exercise (in the long term)	545 (27.3)	718 (35.9)	508 (25.4)	229 (11.5)
Lack of willingness to exercise (in the short/temporary term)	518 (25.9)	745 (37.3)	486 (24.3)	251 (12.6)
Feelings of constant fatigue and lack of energy	488 (24.4)	806 (40.3)	516 (25.8)	190 (9.5)
Competition with activities promoting a sedentary lifestyle (watching TV)	334 (16.7)	777 (38.9)	606 (30.3)	283 (14.2)
Lack of support from the healthcare system (primary care physicians, dietitians, physiotherapists)	332 (16.6)	691 (34.6)	662 (33.1)	315 (15.8)
Lack of skills—I am not physically fit enough	322 (16.1)	667 (33.4)	661 (33.1)	350 (17.5)
Concern due to physical condition (e.g., disability, chronic illness)—my health is not good enough	289 (14.5)	588 (29.4)	620 (31.0)	503 (25.2)
Lack of support from family and friends—I have no one I can exercise with	275 (13.8)	690 (34.5)	640 (32.0)	395 (19.8)
Lack of belief in the effectiveness of physical activity for preventing lifestyle-related diseases	247 (12.4)	611 (30.6)	700 (35.0)	442 (22.1)
Barriers in the built-up environment and geographical isolation (there is no place to exercise, I have no access to fitness clubs, pool)	244 (12.2)	608 (30.4)	675 (33.8)	473 (23.7)
My level of physical activity is currently sufficient	237 (11.9)	668 (33.4)	772 (38.6)	323 (16.2)
Lack of knowledge on current physical activity recommendations	232 (11.6)	688 (34.4)	731 (36.6)	349 (17.5)
Fear of injury	222 (11.1)	577 (28.9)	683 (34.2)	518 (25.9)
Social norms and stigma of “not feeling welcome” (e.g., in a fitness club)	220 (11.0)	563 (28.2)	727 (36.4)	490 (24.5)
Weather conditions	183 (9.2)	649 (32.5)	733 (36.7)	435 (21.8)

**Table 5:** Index of barriers to physical activity according to sociodemographic factors.

Sociodemographic Factors		M(SD) Index Value	Skewness	Dominant	Kruskal–Wallis	*p*
Gender	Women	2.55 (SD = 0.66)	−0.1	3	558.201 *	*p* < 0.001
	Men	2.41 (SD = 0.63)	−0.1	3		
Place of residence	Rural areas	2.54 (SD = 0.65)	−0.1	3	9.177	*p* < 0.05
	City >200 K inhabitants	2.44 (SD = 0.64)	−0.1	3		
	City 200 K to 500 K inhabitants	2.44 (SD = 0.61)	0.1	2		
	City over 500 K inhabitants	2.46 (SD = 0.67)	−0.2	3		
Employment status	Working (employed full-time or self-employed)	2.50 (SD = 0.64)	−0.1	3	20.812	*p* < 0.001
	Student	2.32 (SD = 0.62)	−0.2	2.6		
	Unemployed	2.62 (SD = 0.77)	−0.2	3		
	Retired	2.41 (SD = 0.64)	−0.1	3		
	Disability pensioner	2.51 (SD = 0.66)	−0.3	3		
	Runs a household	2.71 (SD = 0.61)	−0.1	3		
Financial situation	Unsatisfactory	2.69 (SD = 0.69)	−0.3	3	68.166	*p* < 0.001
	Average	2.52 (SD = 0.61)	−0.1	3		
	Satisfactory	2.32 (SD = 0.66)	0.0	2		
Health self-assessment	Unsatisfactory	2.75 (SD = 0.62)	−0.1	2.6	150.220	*p* < 0.001
	Average	2.64 (SD = 0.60)	−0.1	3		
	Satisfactory	2.31 (SD = 0.64)	−0.0	2		
Type of work performed	Blue-collar	2.55 (SD = 0.67)	−0.1	3	7.809	*p* < 0.05
	White-collar	2.45 (SD = 0.63)	−0.2	3		
	Mixed	2.47 (SD = 0.65)	0.0	3		

* Mann–Whitney U test.

## Data Availability

All data generated or analysed during this study are included in this published article. For additional information, please contact the corresponding author.

## Supplementary Materials

The following supporting information can be downloaded at: https://www.mdpi.com/article/10.3390/jcm13010022/s1, Table S1: Barriers limiting healthy diet depending on sex (%, n = 2000). Table S2: Barriers limiting healthy diet depending on age (%, n = 2000). Table S3: Barriers limiting healthy diet depending on education level (%, n = 2000). Table S4: Barriers limiting healthy diet depending on status on the labor market (%, n = 2000). Table S5: Barriers limiting healthy diet depending on the financial situation (%, n = 2000). Table S6: Barriers limiting healthy diet depending on health status (%, n = 2000). Table S7: Barriers limiting healthy diet depending on the type of work performed (%. n = 2000). Table S8: Barriers limiting regular physical activity depending on sex (%, n = 2000). Table S9: Barriers limiting regular physical activity depending on the place of residence (%, n = 2000). Table S10: Barriers limiting regular physical activity depending on the status on the labor market (%, n = 2000). Table S11: Barriers limiting regular physical activity depending on the financial situation (%, n = 2000). Table S12: Barriers limiting regular physical activity depending on health status (%, n = 2000). Table S13: Barriers limiting regular physical activity depending on the type of work performed (%, n = 2000).
