# Supplementary material for: Barriers to Adherence to Healthy Diet and Recommended Physical Activity Perceived by the Polish Population
Source: J Clin Med. 2023 Dec 19;13(1):22. doi: 10.3390/jcm13010022 (PMC10779332; doi:10.3390/jcm13010022)
Supplement: Supplementary file 1 [file jcm-13-00022-s001.zip › jcm-2752127-supplementary.pdf]

QUESTIONNAIRE

Sociodemografic data

1. Gender:
   a) Woman
   b) Man

2. Year of bith ……………………………….

3. In which voivodeship do you live?:
   a) Lower Silesia
   b) Kuyavia-Pomerania
   c) Lublin Voivodeship
   d) Lubusz
   e) Łódź Voivodeship
   f) Lesser Poland
   g) Masovia
   h) Opole Voivodeship
   i) Subcarpathia
   j) Podlaskie Voivodeship
   k) Pomerania
   l) Silesia
   m) Świętokrzyskie Voivodeship
   n) Warmia-Masuria
   o) Greater Poland
   p) West Pomerania

4. What is your place of residence:
   a) Countryside
   b) Town ≤ 200 000
   c) Town 200 000 - 500 000
   d) Town ≥ 500 000

5. What is your education?:
   a) Primary or middle school
   b) Basic vocational
   c) Secondary or post-secondary education
   d) Higher education (Bachelor’s degree and above)

6. What is your employment status
   a) Employed (full-time or self-employed)
   b) Student
   c) Unemployed
   d) Retired
   e) Disability pensioner
   f) Runs a household

7. How do you assess your financial situation:

a) Very bad
b) Bad
c) Average
d) Good
e) Very good

8. How do you assess your health condition:
   a) Very bad
   b) Bad
   c) Average
   d) Good
   e) Very good

9. What type of work do you perform:
   a) White-collar
   b) Blue-collar
   c) Mixed

Barriers

Healthy diet means: consumption of regular, diverse meals, rich in complex carbohydrates, legumes, vegetables, and fruit; while minimizing or avoiding excessive amounts of animal fat, salt, sweets, highly processed foods, and alcohol.

To what extent do the following factors limit your ability to follow a healthy diet/eating healthy every day:

LP.		Definitely limiting	Limiting to a small extent	Rather not limiting	Definitely not limiting
1	Lack of time and competing priorities (work, family, hobbies)				
2	Costs of healthy food				
3	Adjusting habits to a healthier diet				
4	Lack or limited access to good-quality, organically grown and bred products				
5	Difficulties in avoiding unhealthy foods in local community settings or at gatherings (business, family)				
6	Taste of healthy food				
7	Lack of knowledge on current healthy eating recommendations				
8	Lack of belief in the efficacy of healthy diet for preventing lifestyle-related diseases				
9	Unhealthy or erroneous family dietary patterns				
10	Lack of motivation to enact changes				
11	Availability of high-calorie and fast-food products				
12	Lack of support from family and friends				
13	Lack of knowledge and skills regarding the necessary steps to implement healthy lifestyle principles				
14	Lack of support/solutions/proposals from the health care system (primary care physician, dietitian, physiotherapist)				

Regular physical activity means activity performed at least three times a week, amounting to 150-300 minutes per week.

To what extent do the following factors limit your ability to engage in regular physical activity on a daily basis:

LP		Definitely limiting	Limiting to a small extent	Rather not limiting	Definitely not limiting
1	Lack of time and competing priorities (work, family, hobbies)				
2	Competition with activities promoting a sedentary lifestyle (watching TV)				

3	Social norms and stigma of “not feeling welcome” (e.g. in a fitness club)				
4	Barriers in the built-up environment and geographical isolation (*there is no place to exercise, I have no access to fitness clubs, pool*)				
5	Fear of injury				
6	Weather conditions				
7	My level of physical activity is currently sufficient				
8	Lack of willingness to exercise (in the short/temporary term)				
9	Concern due to physical condition (e.g. disability, chronic illness) – *my health is not good enough*				
10	Feelings of constant fatigue and lack of energy				
11	Lack of knowledge on current physical activity recommendations				
12	Lack of belief in the effectiveness of physical activity for preventing lifestyle-related diseases				
13	Lack of motivation to exercise (in the long term)				
14	Lack of skills – *I am not physically fit enough*				
15	Lack of support from the health care system *(primary care physicians, dietitians, physiotherapists)*				
16	Lack of support from family and friends – *I have no one I can exercise with*				

Table S1. Barriers limiting healthy diet depending on sex (%, n=2000)

Barrier	Sex	Definitely limiting	Slightly limiting	Not really limiting	Definitely not limiting	Chi2	p
Costs of healthy food	women	48.2	33.4	14.6	3.7	25.893	p<0.001
	men	37.9	36.8	21.0	4.3		
Lack of support/solutions/proposals from the health care system (primary care doctor, dietitian, physiotherapist)	women	23.4	35.0	29.9	11.7	22.141	p<0.001
	men	15.4	36.1	33.7	14.8		
Lack of access or difficulties in access to good quality products from organic farming and breeding	women	21.1	35.7	32.5	10.7	14.919	p<0.01
	men	15.0	41.7	32.1	11.2		
Lack of support from family, friends and colleagues	women	18.1	32.8	32.5	16.6	19.937	p<0.001
	men	11.3	32.9	37.6	18.2		
Lack of knowledge about current recommendations on healthy diet	women	14.0	34.6	33.0	18.3	13.235	p<0.01
	men	10.6	37.4	37.6	14.5		

Table S2. Barriers limiting healthy diet depending on age (%, n=2000)

Barrier	Age (years)	Definitely limiting	Slightly limiting	Not really limiting	Definitely not limiting	Chi2	p
Lack of time and competing priorities (work, family, hobbies)	18 - 29	28.0	43.5	20.5	8.1	73.868	p<0.001
	30 - 39	33.6	39.3	19.8	7.3		
	40 - 59	26.6	38.7	24.8	10.0		
	≥60	17.7	36.2	27.4	18.7		
Easy access to high-calorie and fast food products	18 - 29	34.2	34.8	21.7	9.3	53.892	p<0.001
	30 - 39	28.4	31.8	28.6	11.2		
	40 - 59	17.7	36.7	32.8	12.8		
	≥60	19.3	33.5	31.5	15.7		

Table S3. Barriers limiting healthy diet depending on education level (%, n=2000)

Barrier	Education level	Definitely limiting	Slightly limiting	Not really limiting	Definitely not limiting	Chi2	p
Lack of knowledge and skills on what steps should be taken to apply the principles of a healthy lifestyle	Primary or secondary school	24.6	32.8	31.1	11.5	26.290	p<0.01
	Basic vocational	20.5	34.9	34.5	10.1		
	Secondary or post-secondary	15.7	35.3	32.6	16.4		
	Higher (Bachelor's degree and above)	11.5	36.5	34.1	18.0		
Lack of belief in the effectiveness of a healthy diet in	Primary or secondary school	18.0	36.1	23.0	23.0	37.767	p<0.001
	Basic vocational	18.7	35.6	33.5	12.2		

preventing lifestyle diseases	Secondary or post-secondary	13.9	34.6	34.4	17.1		
	Higher (Bachelor's degree and above)	9.1	31.5	36.9	22.4		
Lack of knowledge about current recommendations for healthy diet	Primary or secondary school	23.0	44.3	24.6	8.2	38.740	p<0.001
	Basic vocational	16.9	37.8	32.4	12.9		
	Secondary or post-secondary	13.3	37.5	34.5	14.7		
	Higher (Bachelor's degree and above)	9.1	33.0	37.7	20.2		

Table S4. Barriers limiting healthy diet depending on status on the labor market (%, n=2000)

Barrier	Status on the labor market	Definitely limiting	Slightly limiting	Not really limiting	Definitely not limiting	Chi2	p
Costs of healthy food	Working (employed or self-employed)	40.3	37.1	19.6	3.0	235.082	p<0.001
	Student	41.2	36.8	17.6	4.4		
	Unemployed	60.2	24.1	8.4	7.2		
	Retired	45.8	33.3	15.1	5.8		
	Disability pensioner	55.1	26.9	12.8	5.1		
	Head of household	50.0	31.1	14.9	4.1		
Lack of time and competing priorities (work, family, hobbies)	Working (employed or self-employed))	29.7	42.3	21.9	6.1	188.065	p<0.001
	Student	30.9	42.6	19.1	7.4		
	Unemployed	20.5	36.1	21.7	21.7		
	Retired	15.5	31.0	30.3	23.1		
	Disability pensioner	10.3	24.4	30.8	34.6		
	Head of household	27.0	40.5	20.3	12.2		
Adapting habits to a healthier diet	Working (employed or self-employed)	26.1	42.5	26.2	5.2	32.176	p<0.01
	Student	19.1	38.2	29.4	13.2		
	Unemployed	30.1	45.8	12.0	12.0		
	Retired	23.4	47.2	20.9	8.5		
	Disability pensioner	21.8	46.2	23.1	9.0		
	Head of household	21.6	51.4	21.6	5.4		
Easy access to high-calorie and fast food products	Working (employed or self-employed)	24.0	35.9	29.4	10.7	47.119	p<0.001
	Student	32.4	41.2	19.1	7.4		
	Unemployed	25.3	34.9	25.3	14.5		
	Retired	19.1	31.2	31.7	18.0		
	Disability pensioner	12.8	35.9	28.2	23.1		
	Head of household	27.0	20.3	41.9	10.8		

Lack of support/solutions /proposals from the health care system (primary care doctor, dietitian, physiotherapist)	Working (employed or self-employed)	19.2	36.7	31.7	12.3	31.571	p<0.01
	Student	14.7	27.9	39.7	17.6		
	Unemployed	22.9	32.5	26.5	18.1		
	Retired	20.2	31.5	33.9	14.4		
	Disability pensioner	12.8	51.3	17.9	17.9		
	Head of household	29.7	32.4	31.1	6.8		
Lack of access or difficulties in access to good quality products from organic farming and breeding	Working (employed or self-employed)	18.5	38.9	33.0	9.7	42.147	p<0.001
	Student	5.9	39.7	38.2	16.2		
	Unemployed	30.1	31.3	18.1	20.5		
	Retired	15.5	40.7	30.8	13.0		
	Disability pensioner	19.2	35.9	34.6	10.3		
	Head of household	27.0	29.7	37.8	5.4		
Lack of support from family, friends and colleagues	Working (employed or self-employed)	14.8	34.2	34.8	16.2	33.522	p<0.01
	Student	17.6	27.9	41.2	13.2		
	Unemployed	20.5	31.3	32.5	15.7		
	Retired	13.5	29.0	36.0	21.6		
	Disability pensioner	12.8	35.9	21.8	29.5		
	Head of household	18.9	36.5	40.5	4.1		
Lack of belief in the effectiveness of a healthy diet in preventing lifestyle diseases	Working (employed or self-employed)	12.4	33.9	36.3	17.3	40.851	p<0.001
	Student	7.4	17.6	39.7	35.3		
	Unemployed	16.9	39.8	19.3	24.1		
	Retired	12.1	31.9	35.3	20.7		
	Disability pensioner	12.8	42.3	29.5	15.4		
	Head of household	23.0	35.1	28.4	13.5		
Lack of knowledge about current recommendations on healthy diet	Working (employed or self-employed)	12.5	35.4	37.8	14.4	35.180	p<0.01
	Student	7.4	30.9	38.2	23.5		
	Unemployed	20.5	37.3	21.7	20.5		
	Retired	10.1	36.6	33.0	20.2		
	Disability pensioner	12.8	39.7	25.6	21.8		
	Head of household	20.3	39.2	27.0	13.5		

Table S5. Barriers limiting healthy diet depending on the financial situation (%, n=2000)

Barrier	Financial situation	Definitely limiting	Slightly limiting	Not really limiting	Definitely not limiting	Chi2	p
Costs of healthy food	Bad	68.6	21.6	7.3	2.4	210.476	p<0.001
	Mean	45.5	38.3	13.3	2.9		
	Good	26.3	35.3	31.5	7.0		
Lack of motivation to introduce changes	Bad	30.7	42.2	18.5	8.7	33.131	p<0.001
	Mean	27.3	38.9	24.9	8.9		
	Good	23.5	32.0	30.4	14.1		
Adapting habits to a healthier diet	Bad	34.8	40.8	15.7	8.7	44.471	p<0.001
	Mean	25.2	45.8	24.1	4.9		
	Good	20.0	42.1	28.9	9.0		
Easy access to high-calorie and fast food products	Bad	26.5	36.9	23.7	12.9	21.833	p<0.01
	Mean	22.7	36.0	30.5	10.8		
	Good	21.6	30.1	31.5	16.9		
Difficulty avoiding unhealthy food in local community places or during meetings (business, family)	Bad	30.3	36.2	23.0	10.5	29.450	p<0.001
	Mean	20.2	42.2	28.9	8.7		
	Good	17.6	37.4	34.1	11.0		
Lack of support/solutions/proposals from the health care system (primary care doctor, dietitian, physiotherapist)	Bad	26.5	34.5	26.8	12.2	43.458	p<0.001
	Mean	20.2	38.0	31.3	10.5		
	Good	15.0	31.1	35.0	19.0		
Lack of access or difficulties in access to good quality products from organic farming and breeding	Bad	33.4	35.9	21.3	9.4	92.983	p<0.001
	Mean	17.4	42.4	30.4	9.8		
	Good	12.2	32.3	41.6	13.9		
Negative family eating patterns	Bad	26.1	32.8	26.1	15.0	27.216	p<0.001
	Mean	16.6	37.4	31.0	14.9		
	Good	16.5	31.7	31.0	20.9		
Lack of support from family, friends and colleagues	Bad	21.6	36.6	25.8	16.0	45.594	p<0.001
	Mean	15.1	35.1	34.2	15.6		
	Good	11.1	26.4	40.9	21.6		
Lack of knowledge and skills on what steps should be taken to apply the principles of a healthy lifestyle	Bad	22.3	35.9	26.5	15.3	56.232	p<0.001
	Mean	14.8	39.1	33.3	12.8		
	Good	11.5	28.7	37.2	22.6		
Lack of belief in the effectiveness of a healthy diet in preventing lifestyle diseases	Bad	20.2	35.2	28.9	15.7	39.894	p<0.001
	Mean	12.0	36.1	35.1	16.9		
	Good	10.6	27.7	37.7	24.0		
Lack of knowledge about current recommendations on healthy diet	Bad	20.6	35.2	30.0	14.3	49.159	p<0.001
	Mean	11.2	39.7	34.6	14.4		
	Good	10.6	28.7	39.0	21.7		
The taste of healthy food	Bad	17.8	36.6	30.3	15.3	25.678	p<0.001
	Mean	11.2	37.5	34.4	16.9		
	Good	11.5	30.1	35.5	23.0		

Table S6. Barriers limiting healthy diet depending on health status (%, n=2000)

Barrier	Health status	Definitely limiting	Slightly limiting	Not really limiting	Definitely not limiting	Chi2	p
Costs of healthy food	Bad	62.3	22.5	13.1	2.1	61.702	p<0.001
	Mean	45.9	37.3	13.3	3.4		
	Good	37.4	35.6	22.2	4.9		
Lack of motivation to introduce changes	Bad	34.6	38.7	17.8	8.9	42.882	p<0.001
	Mean	30.2	38.8	24.0	7.0		
	Good	22.3	36.0	28.4	13.4		
Lack of time and competing priorities (work, family, hobbies)	Bad	25.7	27.2	25.7	21.5	32.626	p<0.001
	Mean	26.4	42.4	20.8	10.3		
	Good	24.4	38.0	26.2	11.4		
Adapting habits to a healthier diet	Bad	36.1	35.6	22.0	6.3	49.313	p<0.001
	Mean	28.8	46.2	20.2	4.7		
	Good	19.8	43.8	28.0	8.3		
Easy access to high-calorie and fast food products	Bad	27.2	34.0	22.5	16.2	30.335	p<0.001
	Mean	24.7	37.3	29.0	9.0		
	Good	20.5	32.1	31.9	15.4		
Difficulty avoiding unhealthy food in local community places or during meetings (business, family)	Bad	29.3	32.5	23.6	14.7	43.153	p<0.001
	Mean	22.4	44.2	26.8	6.5		
	Good	18.0	37.8	33.0	11.2		
Lack of support/solutions/proposals from the health care system (primary care doctor, dietitian, physiotherapist)	Bad	31.4	33.0	22.5	13.1	72.614	p<0.001
	Mean	22.5	40.2	28.1	9.1		
	Good	14.8	32.0	36.5	16.7		
	Mean						
	Good						
Negative family eating patterns	Bad	23.6	32.5	26.2	17.8	47.642	p<0.001
	Mean	20.0	40.1	28.5	11.4		
	Good	15.1	31.4	32.6	20.8		
Lack of support from family, friends and colleagues	Bad	23.0	30.9	26.2	19.9	54.210	p<0.001
	Mean	17.7	37.1	31.5	13.7		
	Good	11.0	29.7	39.4	19.9		
Lack of knowledge and skills on what steps should be taken to apply the principles of a healthy lifestyle	Bad	20.4	40.3	23.6	15.7	59.736	p<0.001
	Mean	16.6	41.1	31.2	11.2		
	Good	12.4	30.2	37.3	20.1		
Lack of belief in the effectiveness of a healthy diet in preventing lifestyle diseases	Bad	23.0	34.6	27.7	14.7	89.280	p<0.001
	Mean	14.3	40.6	31.9	13.2		
	Good	9.5	27.4	38.9	24.2		
Lack of knowledge about current recommendations on healthy eating	Bad	24.1	32.5	28.3	15.2	78.708	p<0.001
	Mean	14.8	41.2	31.8	12.2		
	Good	8.1	32.1	39.4	20.3		
The taste of healthy food	Bad	24.1	33.0	26.7	16.2	64.416	p<0.001
	Mean	13.9	39.5	32.0	14.5		
	Good	8.4	32.1	37.4	22.1		

Table S7. Barriers limiting healthy diet depending on the type of work performed (%. n=2000)

Barrier	Type of work performed	Definitely limiting	Slightly limiting	Not really limiting	Definitely not limiting	Chi2	p
Lack of motivation to introduce changes	Physical	33.0	36.2	22.1	8.7	19.636	p<0.01
	Mental	23.4	37.3	27.6	11.7		
	Mixed	25.7	38.8	25.7	9.8		
Lack of time and competing priorities (work, family, hobbies)	Physical	29.5	37.0	23.7	9.8	17.938	p<0.01
	Mental	24.8	41.4	22.4	11.4		
	Mixed	22.1	36.4	26.6	14.9		
Lack of knowledge and skills on what steps should be taken to apply the principles of a healthy lifestyle	Physical	21.0	36.6	29.2	13.2	31.196	p<0.001
	Mental	11.6	34.7	35.4	18.3		
	Mixed	14.1	36.2	34.6	15.0		
Lack of belief in the effectiveness of a healthy diet in preventing lifestyle diseases	Physical	18.1	36.2	32.4	13.2	37.495	p<0.001
	Mental	9.4	32.3	36.5	21.9		
	Mixed	12.9	33.0	35.0	19.2		
Lack of knowledge about current recommendations on healthy eating	Physical	17.2	38.6	32.1	12.1	31.732	p<0.001
	Mental	9.7	33.5	37.7	19.1		
	Mixed	12.0	37.1	34.2	16.7		
The taste of healthy food	Physical	16.3	37.7	32.4	13.6	26.125	p<0.001
	Mental	11.0	32.8	34.6	21.5		
	Mixed	10.0	36.8	35.1	18.1		

Table S8. Barriers limiting regular physical activity depending on sex (%, n=2000)

Barrier	Sex	Definitely limiting	Slightly limiting	Not really limiting	Definitely not limiting	Chi2	p
Lack of motivation to exercise	Women	31.1	35.9	22.4	10.5	21.443	p<0.001
	Men	23.0	35.9	28.6	12.5		
No willingness to exercise	Women	29.7	38.2	20.9	11.2	26.473	p<0.001
	Men	21.7	36.2	28.0	14.1		
Feeling constant fatigue and lack of energy	Women	28.7	40.5	21.9	9.0	30.082	p<0.001
	Men	19.7	40.1	30.1	10.1		
Lack of support from the health care system (primary care doctor, dietitian, physiotherapist)	Women	19.9	34.5	31.4	14.2	19.210	p<0.001
	Men	13.0	34.6	34.9	17.4		
Lack of skills (I'm not physically fit enough)	Women	20.3	34.3	30.1	15.3	37.179	p<0.001
	Men	11.4	32.3	36.3	19.9		
Lack of support from family, friends, acquaintances (I have no one to exercise with)	Women	16.7	35.1	30.0	18.2	19.538	p<0.001
	Men	10.5	33.9	34.2	21.4		
Lack of belief in the effectiveness of physical activity in the prevention of lifestyle diseases	Women	14.4	31.4	32.9	21.3	11.712	p<0.01
	Men	10.1	29.6	37.4	23.0		
Barriers in the built environment and	Women	15.2%	30.6%	31.4%	22.8%	20.235	p<0.001
	Men	8.9%	30.2%	36.3%	24.6%		

geographical isolation (I have nowhere to exercise, no access to fitness clubs, swimming pool)							
Atmospheric conditions	Women	10.9%	33.8%	35.1%	20.2%	12.439	p<0.01
	Men	7.2%	31.0%	38.3%	23.5%		

Table S9. Barriers limiting regular physical activity depending on the place of residence (%, n=2000)

Barrier	Place of residence	Definitely limiting	Slightly limiting	Not really limiting	Definitely not limiting	Chi2	p
Lack of belief in the effectiveness of physical activity in the prevention of lifestyle diseases	Village	16.1	28.7	35.6	19.6	23.169	p<0.01
	City < 200.000 citizens	8.6	31.8	36.3	23.3		
	City 200 000 - 500 000 citizens	10.4	31.5	34.4	23.7		
	City < 500 tys. citizens	11.9	31.9	31.6	24.5		
Barriers in the built environment and geographical isolation (I have nowhere to exercise, no access to fitness clubs, swimming pool)	Village	16.8	33.2	30.9	19.1	51.315	p<0.001
	City < 200.000 citizens	7.6	30.7	36.7	25.0		
	City 200 000 - 500 000 citizens	10.1	26.3	38.3	25.3		
	City < 500 tys. citizens	11.3	26.8	31.0	31.0		

Table S10. Barriers limiting regular physical activity depending on the status on the labor market (%, n=2000)

Barrier	Status on the labor market	Definitely limiting	Slightly limiting	Not really limiting	Definitely not limiting	Chi2	p
Lack of time and competing priorities (work, family, hobbies)	Working (employed or self-employed)	34.2	36.3	21.5	8.1	153.068	p<0.001
	Student	33.8	39.7	14.7	11.8		
	Unemployed	22.9	37.3	21.7	18.1		
	Retired	17.5	30.8	29.0	22.7		
	Disability pensioner	10.3	35.9	17.9	35.9		
	Head of household	31.1	44.6	18.9	5.4		
Competition with activities promoting a sedentary lifestyle (watching TV)	Working (employed or self-employed)	17.3	40.2	30.4	12.2	39.904	p<0.001
	Student	14.7	45.6	23.5	16.2		
	Unemployed	18.1	42.2	24.1	15.7		
	Retired	15.5	31.2	35.3	18.0		
	Disability pensioner	12.8	44.9	16.7	25.6		
	Head of household	18.9	45.9	27.0	8.1		
Lack of skills (I'm not physically fit enough)	Working (employed or self-employed)	14.9	33.1	35.1	16.8	52.268	p<0.001
	Student	11.8	23.5	30.9	33.8		
	Unemployed	25.3	39.8	14.5	20.5		

	Retired	15.5	32.1	33.3	19.1		
	Disability pensioner	26.9	42.3	17.9	12.8		
	Head of household	21.6	36.5	35.1	6.8		
Concern due to physical condition (e.g. disability, chronic disease)	Working (employed or self-employed)	12.1	29.6	32.8	25.5	95.128	p<0.001
	Student	8.8	16.2	29.4	45.6		
	Unemployed	16.9	39.8	20.5	22.9		
	Retired	15.1	28.8	31.9	24.3		
	Disability pensioner	42.3	34.6	9.0	14.1		
	Head of household	24.3	24.3	31.1	20.3		
Lack of support from family, friends, acquaintances (I have no one to exercise with)	Working (employed or self-employed)	13.2	35.9	31.8	19.1	31.175	p<0.01
	Student	5.9	29.4	42.6	22.1		
	Unemployed	19.3	33.7	28.9	18.1		
	Retired	14.2	28.8	34.8	22.2		
	Disability pensioner	14.1	42.3	19.2	24.4		
	Head of household	21.6	41.9	25.7	10.8		
Barriers in the built environment and geographical isolation (I have nowhere to exercise, no access to fitness clubs, swimming pool)	Working (employed or self-employed)	10.9	31.8	35.4	21.9	47.953	p<0.001
	Student	11.8	32.4	23.5	32.4		
	Unemployed	19.3	32.5	24.1	24.1		
	Retired	11.0	25.8	35.5	27.6		
	Disability pensioner	16.7	28.2	24.4	30.8		
	Head of household	28.4	32.4	25.7	13.5		
Sufficient level of physical activity in the opinion of respondents	Working (employed or self-employed)	12.4	33.1	39.5	15.1	31.311	p<0.01
	Student	11.8	30.9	30.9	26.5		
	Unemployed	19.3	41.0	25.3	14.5		
	Retired	7.6	32.8	40.7	18.9		
	Disability pensioner	12.8	33.3	37.2	16.7		
	Head of household	18.9	36.5	35.1	9.5		
Fear of injury	Working (employed or self-employed)	9.9	28.1	36.3	25.7	45.362	p<0.001
	Student	5.9	19.1	29.4	45.6		
	Unemployed	15.7	37.3	20.5	26.5		
	Retired	13.0	27.9	33.5	25.6		
	Disability pensioner	15.4	44.9	19.2	20.5		
	Head of household	14.9	29.7	37.8	17.6		

Table S11. Barriers limiting regular physical activity depending on the financial situation (%, n=2000)

Barrier	Financial situation	Definitely limiting	Slightly limiting	Not really limiting	Definitely not limiting	Chi2	p
Lack of motivation to exercise	Bad	35.2	36.6	17.8	10.5	45.213	p<0.001
	Mean	27.9	38.1	24.3	9.7		
	Good	21.9	31.1	31.5	15.5		
No willingness to exercise	Bad	33.8	37.3	16.4	12.5	34.565	p<0.001
	Mean	25.7	39.2	24.5	10.5		
	Good	22.3	33.4	27.8	16.5		
Feeling constant fatigue and lack of energy	Bad	39.0	38.0	13.2	9.8	88.811	p<0.001
	Mean	24.1	43.3	25.4	7.2		
	Good	17.7	35.5	32.9	13.9		
Competition with activities promoting a sedentary lifestyle (watching TV)	Bad	24.4	37.3	25.4	12.9	27.104	p<0.001
	Mean	16.3	40.8	30.2	12.7		
	Good	13.6	35.8	32.9	17.7		
Lack of support from the health care system (primary care doctor, dietitian, physiotherapist)	Bad	28.2	31.4	27.5	12.9	82.593	p<0.001
	Mean	16.7	38.2	32.5	12.6		
	Good	10.6	28.9	37.0	23.5		
Lack of skills (I'm not physically fit enough)	Bad	27.2	36.6	20.9	15.3	71.336	p<0.001
	Mean	15.2	36.3	32.9	15.6		
	Good	12.3	25.9	39.5	22.3		
Concern due to physical condition (e.g. disability, chronic disease)	Bad	28.6	34.8	20.6	16.0	100.671	p<0.001
	Mean	13.4	31.7	30.4	24.5		
	Good	9.6	22.1	37.4	31.0		
Lack of support from family, friends, acquaintances (I have no one to exercise with)	Bad	22.3	34.5	25.4	17.8	46.760	p<0.001
	Mean	14.0	36.6	32.0	17.5		
	Good	9.0	30.4	35.3	25.2		
Lack of belief in the effectiveness of physical activity in the prevention of lifestyle diseases	Bad	19.9	30.3	31.4	18.5	39.255	p<0.001
	Mean	12.1	33.3	34.0	20.6		
	Good	9.0	25.2	38.8	27.0		
Barriers in the built environment and geographical isolation (I have nowhere to exercise, no access to fitness clubs, swimming pool)	Bad	22.0	30.3	27.5	20.2	45.143	p<0.001
	Mean	10.9	32.7	34.4	22.0		
	Good	9.9	25.9	35.5	28.7		
Sufficient level of physical activity in the opinion of respondents	Bad	20.6	33.1	30.3	16.0	36.339	p<0.001
	Mean	10.5	35.0	40.1	14.4		
	Good	10.1	30.4	39.8	19.7		
Lack of knowledge about current recommendations regarding physical activity	Bad	18.1	35.5	28.9	17.4	40.449	p<0.001
	Mean	11.2	37.0	37.0	14.9		
	Good	9.2	28.7	39.5	22.6		
Fear of injury	Bad	17.4	35.9	27.2	19.5	49.137	p<0.001
	Mean	10.5	30.5	34.9	24.1		
	Good	9.0	22.1	36.2	32.7		
Social norms and stigma "I don't feel welcome" (e.g. in a fitness club)	Bad	17.1	28.2	32.4	22.3	30.707	p<0.001
	Mean	10.2	30.4	37.3	22.1		
	Good	9.6	23.7	36.3	30.4		
Atmospheric conditions	Bad	13.9	37.3	29.6	19.2	31.466	p<0.001
	Mean	8.4	34.5	36.6	20.4		
	Good	8.2	25.9	40.2	25.7		

Table S12. Barriers limiting regular physical activity depending on health status (%, n=2000)

Barrier	Health status	Definitely limiting	Slightly limiting	Not really limiting	Definitely not limiting	Chi2	p
Lack of time and competing priorities (work, family, hobbies)	Bad	29.5	39.2	22.2	9.2	34.191	p<0.001
	Mean	27.2	30.4	18.8	23.6		
	Good	28.9	33.4	23.9	13.8		
Lack of motivation to exercise	Bad	39.8	30.4	19.9	9.9	59.268	p<0.001
	Mean	30.5	39.2	22.7	7.6		
	Good	22.1	34.2	28.8	14.9		
No willingness to exercise	Bad	36.6	33.5	16.8	13.1	54.062	p<0.001
	Mean	29.6	39.5	22.4	8.5		
	Good	20.7	36.1	27.3	15.9		
Feeling constant fatigue and lack of energy	Bad	50.3	30.4	15.7	3.7	165.541	p<0.001
	Mean	28.6	44.5	21.6	5.3		
	Good	15.9	38.7	31.3	14.1		
Competition with activities promoting a sedentary lifestyle (watching TV)	Bad	21.5	34.0	23.6	20.9	57.096	p<0.001
	Mean	19.5	43.3	28.5	8.7		
	Good	13.4	36.1	33.1	17.4		
Lack of support from the health care system (primary care doctor, dietitian, physiotherapist)	Bad	30.4	30.9	29.8	8.9	114.325	p<0.001
	Mean	18.3	42.3	29.6	9.8		
	Good	12.5	28.8	36.7	22.1		
Lack of skills (I'm not physically fit enough)	Bad	41.4	34.0	18.3	6.3	182.280	p<0.001
	Mean	18.1	38.2	31.3	12.5		
	Good	9.6	29.2	37.4	23.9		
Concern due to physical condition (e.g. disability, chronic disease)	Bad	47.6	31.9	16.2	4.2	383.451	p<0.001
	Mean	16.1	37.8	31.8	14.3		
	Good	6.6	21.8	33.2	38.3		
Lack of support from family, friends, acquaintances (I have no one to exercise with)	Bad	23.0	36.1	25.1	15.7	77.078	p<0.001
	Mean	16.5	39.6	29.6	14.3		
	Good	9.7	29.9	35.4	25.1		
Lack of belief in the effectiveness of physical activity in the prevention of lifestyle diseases	Bad	25.7	31.9	26.7	15.7	99.462	p<0.001
	Mean	14.9	35.6	32.5	17.0		
	Good	7.6	26.0	38.7	27.6		
Barriers in the built environment and geographical isolation (I have nowhere to exercise, no access to fitness clubs, swimming pool)	Bad	19.4	31.9	25.1	23.6	52.395	p<0.001
	Mean	14.9	33.8	32.5	18.8		
	Good	8.5	27.2	36.5	27.7		
Sufficient level of physical activity in the opinion of respondents	Bad	24.6	27.2	28.8	19.4	65.149	p<0.001
	Mean	13.0	37.3	37.6	12.1		
	Good	8.4	31.3	41.4	18.9		
Lack of knowledge about current recommendations regarding physical activity	Bad	23.0	29.3	33.0	14.7	105.322	p<0.001
	Mean	14.4	41.5	32.1	12.0		
	Good	7.0	29.5	41.0	22.6		
Fear of injury	Bad	26.7	33.5	25.1	14.7	146.661	p<0.001
	Mean	12.0	35.2	35.0	17.8		
	Good	7.3	22.7	35.2	34.9		
Social norms and stigma "I don't feel welcome" (e.g. in a fitness club)	Bad	16.8	24.6	33.0	25.7	63.115	p<0.001
	Mean	13.9	32.1	36.6	17.3		
	Good	7.4	25.5	36.8	30.3		
Atmospheric conditions	Bad	15.2	33.0	34.0	17.8	57.874	p<0.001
	Mean	10.4	38.3	34.9	16.4		

	Good	6.9	27.4	38.6	27.0		

Table S13 Barriers limiting regular physical activity depending on the type of work performed (%, n=2000)

Barrier	Type of work	Definitely limiting	Slightly limiting	Not really limiting	Definitely not limiting	Chi2	p
Lack of belief in the effectiveness of physical activity in the prevention of lifestyle diseases	Physical	17.2	31.9	32.6	18.3	22.005	p<0.01
	Mental	10.3	29.6	35.9	24.2		
	Mixed	10.9	30.8	35.9	22.5		
